# A High Circulating Tumor Cell Count in Portal Vein Predicts Liver Metastasis From Periampullary or Pancreatic Cancer: A High Portal Venous CTC Count Predicts Liver Metastases

**DOI:** 10.1097/MD.0000000000003407

**Published:** 2016-04-22

**Authors:** Yu Wen Tien, Hsun-Chuan Kuo, Be-Ing Ho, Ming-Chu Chang, Yu-Ting Chang, Mei-Fang Cheng, Huai-Lu Chen, Ting-Yung Liang, Chien-Fang Wang, Chia-Yi Huang, Jin-Yuh Shew, Ying Chih Chang, Eva YHP Lee, Wen-Hwa Lee

**Affiliations:** From the Department of Surgery (YWT, H-CK, B-IH); Department of Internal Medicine (M-CC, Y-TC); Department of Nuclear Medicine, National Taiwan University Hospital (M-FC); Genomics Research Center, Academia Sinica, Taipei (H-LC, T-YL, C-FW, C-YH, J-YS, YCC, EYL, W-HL); and Institute of Clinical Medicine, China Medical University, Taiwan (EYL, W-HL).

## Abstract

Circulating tumor cells (CTCs) released from a periampullary or pancreatic cancer can be more frequently detected in the portal than the systemic circulation and potentially can be used to identify patients with liver micrometastases. Aims of this study is to determine if CTCs count in portal venous blood of patients with nonmetastatic periampullary or pancreatic adenocarcinoma can be used as a predictor for subsequent liver metastases. CTCs were quantified in portal and peripheral venous blood samples collected simultaneously during pancreaticoduodenectomy in patients with presumed periampullary or pancreatic adenocarcinoma without image-discernible metastasis. Postoperatively patients were monitored for liver metastasis by abdominal magnetic resonance imaging or computed tomography every 3 months for 1 year. Sixty patients with a pathological diagnosis of periampullary or pancreatic adenocarcinoma were included in the study. Multivariate analysis indicated that portal CTC count was a significant predictor for liver metastases within 6 months after surgery. Eleven of 13 patients with a high portal CTCs count (defined as >112 CMx Platform estimated CTCs in 2 mL blood) developed liver metastases within 6 months after surgery. In contrast, only 6 of 47 patients with a low portal CTC count developed liver metastases (*P* < 0.0001). A value of 112 CMx Platform estimated CTCs had 64.7% sensitivity and 95.4% specificity to predict liver metastases within 6 months after surgery. We concluded that a high CTC count in portal venous blood collected during pancreaticoduodenectomy in patients with periampullary or pancreatic adenocarcinoma without metastases detected by currently available imaging tools is a significant predictor for liver metastases within 6 months after surgery.

## INTRODUCTION

Current pretreatment assessment of cancer is based on contrast-enhanced computed tomography (CT) or magnetic resonance imaging (MRI). Although CT and MRI provide us anatomic delineation of the lesion, they are insufficient to accurately determine the presence or absence of metastases. Positron emission tomography (PET) is also unable to detect micrometastatic disease because of limited resolution of 4 to 10 mm.^1,2^ Operation on patients with image-indiscernible micrometastasis will subject them to operative risk and delay in systemic therapy without any benefit in survival. One method of screening out micrometastatic disease can avoid an unnecessary or even harmful operation.

Animal studies have shown that the risk of metastasis formation is proportional to the amount of injected tumor cells.^3^ Therefore, patients with abundant circulating tumor cells (CTCs) may also have a higher risk of metastasis; hence, the enumeration of CTCs may help to identify carcinoma patients with image-indiscernible micrometastases. However, previous study have indicated that CTCs are rarely detected in the peripheral venous blood of patients.^4^ The rarity of CTCs in the peripheral venous blood of patients with nonmetastatic carcinoma greatly limits its use as a predictor for metastasis.

The diameter of CTCs is around 25 μm which is far too large to allow them to pass through the capillaries (∼8 μm diameter).^5^ It has been demonstrated in animal models that most radiolabeled tumor cells injected into a vein are trapped in the capillary beds of the first target organ, and few are detectable in the peripheral blood.^6^ It has recently been shown in colorectal cancer patients that CTCs can be detected at a higher rate and at a higher number in tumor drainage (mesenteric) blood than in peripheral venous blood.^7^ The portal vein drains periampullary and pancreatic tissues. Theoretically CTCs will be more easily detected in portal than in peripheral venous blood of patients with periampullary or pancreatic adenocarcinoma. Hence, enumeration of CTCs in portal venous blood may potentially be an early indication of liver micrometastases in patients with periampullary and pancreatic cancer.

To test this hypothesis, we evaluated CTCs in portal and peripheral venous blood samples collected simultaneously during pancreaticoduodenectomy (PD) in patients with presumed periampullary or pancreatic adenocarcinoma without liver metastasis identified on preoperative images. CTCs counts in portal venous blood samples were correlated with the appearance of liver metastases within 6 months after surgery to determine if they can be used as a predictor for liver metastases.

## METHODS

### Patients

Patients in whom PD was planned for presumed periampullary or pancreatic adenocarcinoma without metastasis as determined by CT and/or MRI at the Department of Surgery, National Taiwan University Hospital between June 2013 and August 2014 were eligible for inclusion in this prospective study. Patients with a history of another malignancy who was diagnosed or treated within the past 5 years, those who received neo-adjuvant chemotherapy or radiotherapy, and those with an admission for a diagnosis of acute or chronic pancreatitis within the 2 months before surgery were also excluded. The study protocol was approved by the Ethics Committee of the National Taiwan University Hospital (201303029RINC) and all patients gave written informed consent to donate blood samples for research.

### Preoperative Assessment

The 7th edition of the American Joint Committee of Cancer (AJCC) TNM Classification of Malignant Tumors was used for clinical tumor staging. In addition to measurement of serum carbohydrate antigen 19-9 (CA19-9) and carcinoembryonic antigen (CEA), all patients received FDG-PET-CT before surgery and only patients in whom no metastasis was shown by FDG-PET-CT underwent operation.

### Blood Sampling and Quantification of CTCs

After entering peritoneal cavity, we inspected and palpated the liver and peritoneal cavity to identify any possible metastasis. Biopsy was performed for any suspicious lesions and PD was abandoned if a frozen section of intraoperative specimens were positive for metastatic adenocarcinoma. PD was started with isolation and division of the common bile duct at a site just proximal to its junction with the cystic duct, and then the portal vein was exposed. Three milliliters of blood was then collected from the portal vein by direct puncture with a syringe with a Fr.-21-needle before manipulation of the tumor. At time of portal venous blood collection, 3 mL peripheral venous blood was also collected. Blood samples were transferred into 5 mL ethylenediaminetetraacetic acid vacutainer tubes (BD, K2E), maintained at room temperature, and processed within 4 hours. CTC collection and analysis with the cells in maximum (CMx) Platform was performed in an operator-blinded fashion. Briefly, the CMx Platform consists of a chaotic mixing microfluidic chip coated with an anti-EpCAM (EpAb4-1) conjugated lipid bilayer film and a second membrane chip to collect cells for imaging analysis.^8^ Using spiked 5 to 1000 HCT116 cancer cell line as a control, the capture efficiency is linear and on the average of 92% ± 1% (n = 852). For the samples, 2 mL of whole blood was processed. After capturing in the microfluidic chip, the bounded cells were eluted and concentrated on an ∼1 cm diameter planar TTTP membrane (2 μm pore size, Millipore TTTP02500) to carry out the immunofluorescence staining.^9^ Antibodies against pancytokeratin (panCK) (AbCAM, cat.ab9377) and goat antirabbit Alexa 647 (Life Technologies) were used for positive selection of tumor cells; an antibody against CD45 conjugated with FITC (DAKO, clone no. T29/33) was used for leukocyte exclusion; and 4′,6-diamidino-2-phenylindole (DAPI) was used to stain the nuclei. The presence of CTCs was decided by trained operators. Only panCK^+^/CD45^−^/DAPI^+^ cells with the correct cell morphology were defined as CTCs. To establish a cutoff value, 39 volunteers with no known cancer history were recruited. Using 95% as a cutoff, we could generate a cutoff value of 13% or 98% specificity (only 1 out of 39 donors has >13 CTC count).

### Adjuvant Therapy

After surgery, adjuvant chemotherapy was recommended to all patients with a final pathological diagnosis of pancreatic ductal adenocarcinoma (PDAC), ampullary cancer, distal common bile duct cancer, and duodenal cancer. After explanation, the patients themselves decided whether or not to receive adjuvant chemotherapy. In addition, patients decided the form of chemotherapy, either oral titanium silicate (TS)-1 or a gemcitabine-based formula.

### Postoperative Follow-Up

Postoperatively patients were followed up every 3 months for 1 year with serum CA19-9 level, chest radiography, and abdominal MRI (for patients with an MRI as a preoperative staging image) or CT (for patients with a CT as a preoperative staging image). Liver metastases were diagnosed as the presence of a new low-density mass in the liver that was enlarging, or the appearance of a new low-density mass on repeat short-term cross-sectional imaging. Radiographic findings consistent with liver metastases were considered adequate proof of liver metastases, and pathologic confirmation was not obtained.

### Statistical Analysis

We presumed that liver micrometastases present at time of surgery would become detectable by CT or MRI within 6 months after surgery and liver metastases detected at more than 6 months after surgery may have originated from primary residual disease. Therefore, the primary outcome of interest was the appearance of liver metastases within 6 months after surgery.

Statistical analysis was performed using the R 3.1.2 software (R Foundation for Statistical Computing, Vienna, Austria) and a 2-sided *P* value ≤0.05 was considered statistically significant. Continuous variables were expressed by mean ± standard deviation (SD), and categorical variables were presented by frequency and percentage. In univariate analysis, differences in the distributions of continuous variables and categorical variables between the patients with and without liver metastasis within 6 months after surgery were examined using the Wilcoxon rank-sum test and Fisher exact test, respectively. Multivariate analysis was conducted by fitting logistic regression model to estimate the adjusted effects of risk factors, prognostic factors, or predictors on the risk of liver metastasis.

Simple and multiple generalized additive models (GAMs)^10,11^ were fitted to detect nonlinear effects of continuous covariates and identify appropriate cutoff points for discretizing continuous covariates during the stepwise variable selection procedure. A receiver operating characteristic (ROC) curve for liver metastases within 6 months after surgery was created for the validation dataset. The estimated area under the ROC curve (also called the c statistic) ≥0.7 suggests an acceptable level of discrimination power.

## RESULTS

### Study Population

Between June 2013 and August 2014, 70 patients were enrolled prospectively into the study. Two patients were excluded from surgery because of liver metastases identified by FDG-PET-CT. Two operations were converted to biliary bypass because of the intraoperative finding of liver metastases in 1 and peritoneal seeding in the other patient. Therefore, CTCs studies were performed in 66 patients in whom PD was performed. Of the 66 patients having PD, the final pathological diagnosis was 42 PDAC, 15 ampullary cancers, 3 CBD cancers, 1 duodenal cancer, 1 grade 3 neuroendocrine tumor, 2 chronic pancreatitis, and 2 benign neoplasms. One patient with PDCA died of cerebral infarction 2 months after surgery and was excluded from the analysis. The remaining 60 patients with a final pathological diagnosis of periampullary cancer (41 PDAC, 15 ampullary cancer, 1 duodenal CA, and 3 CBD cancers) were put into analysis. The clinicopathological features of these 60 patients are listed in Table 1.

**TABLE 1:**
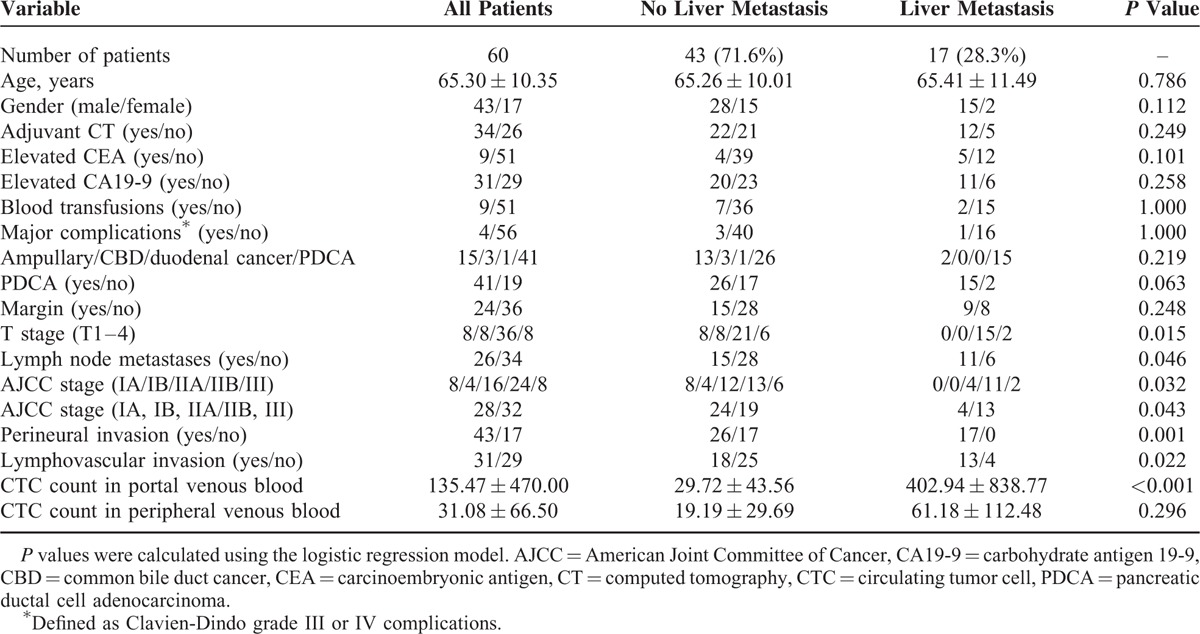
Clinicopathological Characteristics of 60 Studied Patients and Univariate Analysis of Factors Correlated With Liver Metastases Within 6 Months After Operation

### Portal Venous Blood Sample Collection Safety

The results showed that portal venous blood could be safely sampled during surgery by direct puncture using a Fr.-21-needle (PrecisionGlide Needle 21G 1 1/2 TW; BD Becton, Dickinson and company [0.8 mm × 38 mm]). Bleeding stopped after digital compression in 65 of 66 patients and only 1 patient required one 6–0 prolene suture to stop the bleeding.

### Paired Comparison of CTC Number Between Central and Portal Venous Blood Samples

CTCs were detected at a higher rate (35 [58.3%] vs 24 [40%], *P* = 0.0098) and at a significantly higher number (mean, 230.1 vs 71.7; median, 60.0 vs 40.5, *P* = 0.0002) in portal than in peripheral venous blood of 60 patients with periampullary or pancreatic carcinoma (Table 2). There was no difference in CTC detection either in portal or peripheral venous blood among patients with different stages of disease (Table 2). Stratified by pathologic type, CTCs were also detected at a higher rate (24 [58.5%] vs 16 [39.0%], *P* = 0.0269) and at a significantly higher number (mean, 313.4 vs 92.0; median, 116.5 vs 52.0, *P* = 0.0013) in portal than in peripheral venous blood of 41 patients with PDAC (Table 3).

**TABLE 2:**
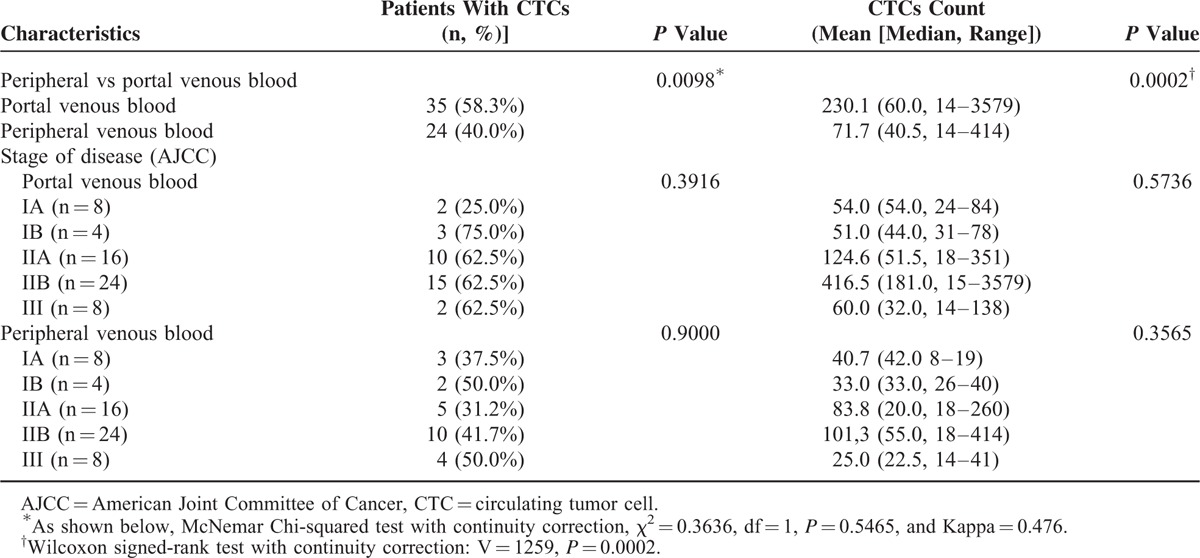
Paired Comparison of Circulating Tumor Cell Detection in Portal and Peripheral Venous Blood Samples of 60 Patients with Periampullary or Pancreatic Adenocarcinoma

**TABLE 3:**
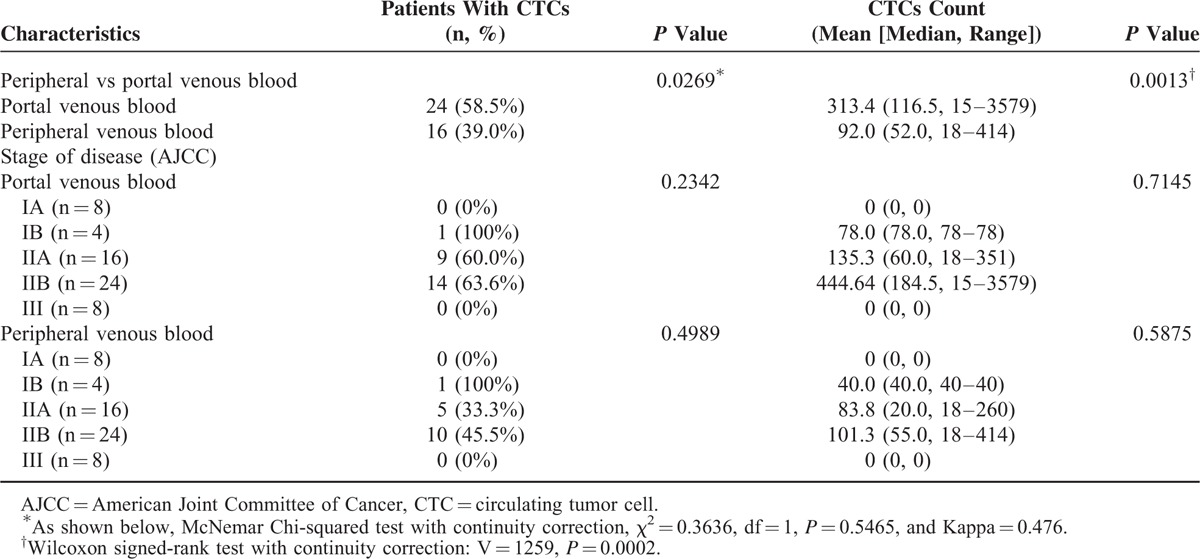
Paired Comparison of Circulating Tumor Cell Detection in Portal and Peripheral Venous Blood Samples of 41 Patients With Pancreatic Adenocarcinoma

### Association of Clinicopathological Variables and CTC Count in Peripheral and Portal Venous Blood With Development of Liver Metastasis Within 6 Months After Surgery

Abdominal MRI or CT performed at 3 months after surgery detected liver metastases in 11 of 60 patients, 5 with and 6 without local recurrence. Of the remaining 49 patients, cross-section images performed at 6 months after surgery revealed liver metastases in 6 patients (5 with and 1 without local recurrence) and local recurrence without liver metastasis in 6 patients. Of the remaining 43 patients without liver metastases detected within 6 months after surgery, abdominal CT or MRI at 9 months after surgery revealed liver metastases with local recurrence in 1 patient and local recurrence without liver metastasis in 1 patient. Of the 42 patients without liver metastases detected within 9 months after surgery, abdominal CT or MRI at 12 months after surgery revealed liver metastases and local recurrence in 1 patient and local recurrence without liver metastasis in 2 patients. Therefore, a total of 17 (28.3%) patients had liver metastases within 6 months after surgery. Thirteen of 17 patients with liver metastases detected within 6 months after operation died at 4, 5, 5, 5, 5, 6, 7, 10, 11, 11, 12, 14, and 16 months after surgery.

Univariate analysis showed higher tumor T-stage (*P* = 0.015), lymph node metastases (*P* = 0.046), AJCC stage (IIb or III, *P* = 0.043), perineural invasion (*P* = 0.001), lymphovascular invasion (*P* = 0.022), and CTC count in portal venous blood (*P* < 0.001, Table 1) but not adjuvant chemotherapy, blood transfusions, or high Clavien-Dindo (III or IV) complications are risk factors for liver metastases within 6 months after surgery. Multivariate analysis of the predictors of liver metastasis by fitting multiple logistic regression models with the stepwise variable selection method showed CTC count in portal venous blood (*P* = 0.0019, Table 4) and lymphovascular invasion (*P* = 0.0408) were significant predictors of liver metastases within 6 months after PD. The cutoff value of 112 CMx Platform evaluated CTCs in 2 mL portal venous blood was estimated by the simple GAM of liver metastasis. Eleven of 13 patients with a CTC count more than 112 CMx Platform estimated CTCs in 2 mL portal venous blood developed liver metastases within 6 months after surgery. In contrast, 6 of 47 patients with a low CTC count (defined as ≤112 CTCs in 2cc portal venous blood) developed liver metastases. A value of 112 CMx Platform estimated CTCs had 64.7% sensitivity and 95.4% specificity to predict liver metastases within 6 months after surgery. Of the 41 patients with PDAC, 15 (36.6%) had liver metastases within 6 months after surgery. Univariate analysis showed CTC count in portal venous blood (*P* = 0.002, Table 5) is a risk factor for liver metastases within 6 months after surgery. Multivariate analysis of the predictors of liver metastasis in 41 PDAC patients showed CTCs count in portal venous blood (*P* = 0.0042, Table 6) was the only significant predictor of liver metastases within 6 months after surgery. Ten of 12 patients with a CTC count more than 112 CMx Platform estimated CTCs in 2 mL portal venous blood developed liver metastases within 6 months after surgery. In contrast, 5 of 29 patients with a low CTC count (defined as ≤112 CTCs in 2cc portal venous blood) developed liver metastases. A value of 112 CMx Platform estimated CTCs had a 64.7% sensitivity and 95.4% specificity to predict liver metastases within 6 months after surgery in 41 patients with PDAC.

**TABLE 4:**
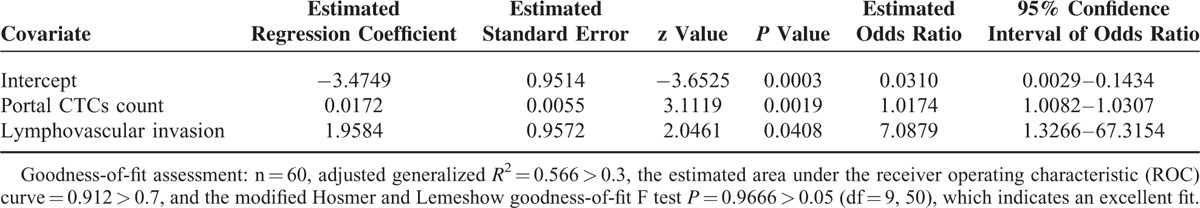
Multivariate Analysis of the Predictors of Liver Metastasis by Fitting Multiple Logistic Regression Models With the Stepwise Variable Selection Method in 60 Patients With Peri-Ampullary or Pancreatic Adenocarcinoma

**TABLE 5:**
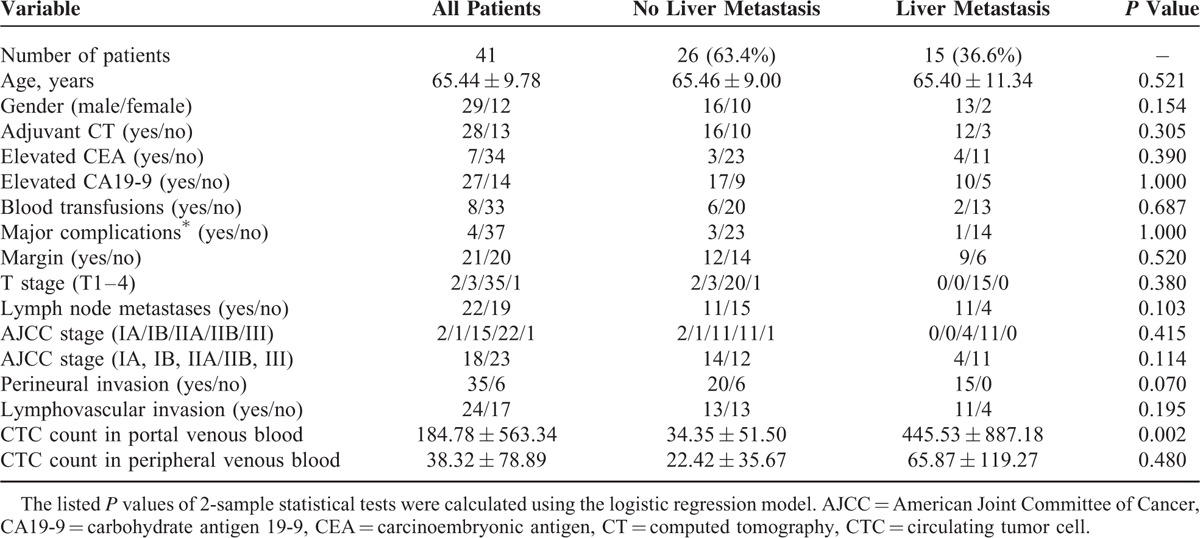
Clinicopathological Characteristics of 41 Pancreatic Ductal Cell Adenocarcinoma Patients and Univariate Analysis of Factors Correlated With Liver Metastases within 6 Months After Operation

**TABLE 6:**
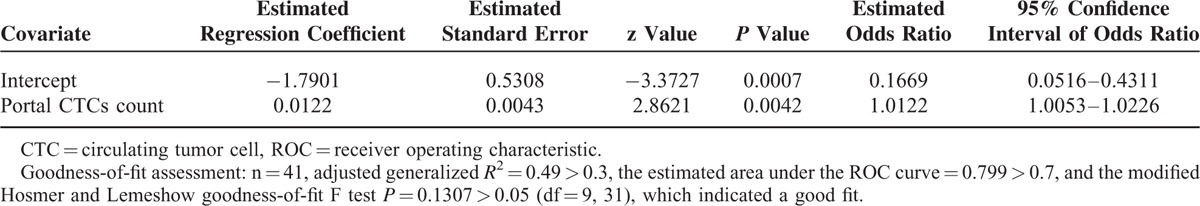
Multivariate Analysis of the Predictors of Liver Metastasis by Fitting Multiple Logistic Regression Model With the Stepwise Variable Selection Method in 41 Patients with Pancreatic Ductal Adenocarcinoma

## DISCUSSION

This study proved that CTCs could be detected at a higher rate (58.3% vs 40.0%, *P* = 0.0098) and a higher count (mean, 230.1 vs 71.7, *P* = 0.0002) in portal than in peripheral venous blood of patients with periampullary and pancreatic adenocarcinoma without image-discernible metastasis. Recently, Catenacci et al^12^ performed endoscopic ultrasound (EUS) guided transhepatic portal venous sampling in 18 patients with pancreaticobiliary carcinoma and also confirmed significantly higher CTC count in portal than in peripheral venous blood. The liver appears to be a sieve for CTCs, and fewer enter the peripheral venous blood thus confirming the establishment of liver metastases being dependent on the first-pass trapping of tumor cells in the liver.^3,6^

Patterns of recurrence after curative resection of carcinoma will depend on the frequency and intensity of surveillance. Increasing the frequency and intensity will detect more preclinical recurrences at an earlier time after resection of pancreatic cancer. In this study, we followed up patients every 3 months with abdominal CT or MRI for 1 year, and detected liver metastases in 17 patients within 6 months after surgery and in only 2 patients at more than 6 months after surgery (12 months). Most importantly, 11 of 13 patients with a high CTC count (defined as >112 CMx Platform estimated CTCs in 2 mL blood) had liver metastases and all of the 11 patients had liver metastases within 6 months after surgery. The fact that liver metastases developed so soon after surgery in patients with a high portal venous CTC count also suggests of the presence of micrometastases at time of operation.

Twenty one (60%) of 35 patients with CTCs detected in the portal venous blood did not develop liver metastases within 6 months after surgery. Therefore, detection of CTCs in portal venous blood does not necessarily mean subsequent development of liver metastasis. However, the risk of developing liver metastases after surgery was significantly proportional to the CTC count in portal venous blood sample collected during surgery. Multivariate analysis showed that a high CTC count in portal, but not in peripheral venous blood, was a significant predictor for liver metastases within 6 months after surgery. In spite of correlation between the detection of CTC in the portal and peripheral blood samples, lower detection rate and count may account for the failure of CTC count in peripheral venous blood as a predictor for liver metastasis.

Although the CTC count in portal venous blood sampled during surgery can predict early liver metastases after resection, but it cannot avoid an unnecessary operation. However, it can be used as a guide for the selection of patients for adjuvant therapy. Our study showed the positive predictive value of a high CTC count in portal venous blood for liver micrometastasis is 84.6% (11 of 13 patients) and the negative predictive value of a low CTC count in portal venous blood is 87.2%. Therefore, it will be justified to begin adjuvant therapy in patients with a high CTC count in portal venous blood because they are at high risk for liver metastases. Besides, Catenacci et al^12^ recently reported EUS-guided transhepatic portal venous sampling is feasible and safe. Thakrar and Madoff^13^ also reported the safety of percutaneous transhepatic portal vein embolization with a large-caliber catheter before major hepatectomy. Both of these nonoperative techniques can collect blood from the intrahepatic portal vein for the evaluation of CTC count before operation. Information obtained before operation potentially can be used as a guide to select patients with a resectable periampullary or pancreatic carcinoma for neoadjuvant therapy and morbidity of operation can be avoided.

In addition to enumeration of CTCs, researchers have attempted to characterize metastasis-initiating cells (MICs) as prognostic indicators.^14,15^ However, mathematical models of the metastatic process have indicated that it is impractical to detect circulating MICs in a single blood sample because the number of CTCs in the blood stream far exceeds the number of metastatic lesions in patients, indicating that the vast majority CTCs die in the bloodstream, with only an extremely small fraction representing viable MICs. Therefore, the chances of detecting MICs in one blood test are extremely small. The other obstacle to the detection of MICs is the lack of suitable markers. Reported markers for MICs included CD44^+^/CD7^+^/Met^+^/CD45^−^, and dual epithelial and mesenchymal markers.^14,15^ As stated before MICs comprise only an extremely small fraction of CTCs and theoretically will be more difficult to be detected in the peripheral venous blood of patients with nonmetastatic cancer. Baccelli et al^14^ tried to characterize MICs at a functional level, that is, by using a xenograft system. Interesting, the injection of blood samples depleted of hematopoietic cells from 106 metastatic breast cancer patients with less than 1000 CellSearch evaluated CTCs into the femoral medullar cavity of immunocompromised mice did not lead to any metastatic growth of human tumor cells within 15 months after transplantation. In contrast, blood samples from 3 of 4 patients with more than 1109 CellSearch evaluated CTCs per 7.5 mL blood led to multiple bone, lung, and liver metastases within 6 to 12 months after transplantation. A recent report of patients with small-cell lung cancer also showed that in vivo assays require a very high CTC yield in transplanted blood samples.^16^ These findings demonstrate that functional MICs exist only in blood with a high CTC count. Therefore, blood with a high CTC count will have more MICs and a greater chance to form metastases, which is consistent with our finding that a high portal venous blood CTC count suggests of presence of micrometastases and predicts early liver metastases after surgery.

Five of 29 patients with a low portal venous blood CTC count (defined as ≦112 CMx Platform estimated CTCs in 2 mL blood) developed liver metastases after surgery. This may be explained by intermittent and heterogeneous shedding of tumor cells. Furthermore, we captured only highly expressed EpCAM cells and undetected EpCAM-negative or EpCAM-depleted CTCs might account for these liver metastases. Indeed, recent data have demonstrated that disseminating tumor cells downregulating the expression of epithelial-specific proteins via epithelial-to-mesenchymal transition, which might be missed by EPCAM-based isolation methods, play a role during the initiation of metastasis.^17,18^

In conclusion, we enumerated the CTC count in portal venous blood during PD of 60 patients with periampullary or pancreatic head adenocarcinoma without metastasis detected by currently available imaging tools including FDG-PET-CT. CTC count in portal venous blood is a significant predictor for liver metastases within 6 months after surgery and can be used as a guide for adjuvant therapy.
